# COVID-19-Related Incidental Pancreatitis Detected on FDG-PET Scan

**DOI:** 10.7759/cureus.31730

**Published:** 2022-11-21

**Authors:** Zeeshan Qurban, Damian Mullan

**Affiliations:** 1 Medical Oncology, The Christie NHS Foundation Trust, Manchester, GBR; 2 Interventional Radiology, The Christie NHS Foundation Trust, Manchester, GBR

**Keywords:** covid-19 vaccine complication, classic hodgkin lymphoma, 18f-fluorodeoxyglucose positron emission tomography (18f-fdg pet), incidental radiological finding, acute pancreatitis, covid 19

## Abstract

Although severe acute respiratory syndrome coronavirus 2 (SARS-CoV-2)/coronavirus disease 2019 (COVID-19) infection predominantly affects the respiratory system, it has also been found to be responsible for several gastrointestinal effects due to its capability to attack angiotensin-converting enzyme (ACE) type 2 cells in various parts of the body. Several cases of radiologically confirmed thyroiditis, axillary lymphangitis, and acute pancreatitis related to COVID-19 infection have been reported, which seem to arise from a direct cytotoxic effect of the virus itself. This case report presents an incidental 18-fluoro-2-deoxyglucose (FDG) positron emission tomography (PET) computed tomography (CT) finding of mild pancreatic inflammation/pancreatitis in an otherwise asymptomatic patient undergoing routine imaging as part of the staging process following stem cell transplant, who had recently recovered from a severe form of COVID infection. This case highlights the fact that COVID can trigger insidious inflammatory processes in a variety of organs often remaining clinically undetectable until resultant end-organ damage causes incipient clinical symptoms.

## Introduction

Severe acute respiratory syndrome coronavirus 2 (SARS-CoV-2) is the causative pathogen of the recent pandemic of coronavirus disease 2019 (COVID-19) and has caused more than 179 million infections and 3.8 million deaths worldwide [1].

In addition to well-documented effects on the respiratory system, several sequelae on the gastrointestinal, cardiac, immune, haematological, endocrine and renal systems have also been demonstrated. Radiological imaging, particularly computed tomography (CT) chest has proved vital in the diagnosis, severity scoring and follow-up of the respiratory effects of COVID-19. Sequelae of COVID-19 have been discovered on scans performed for other medical conditions unrelated to COVID-19 [2]. 

This article presents a case of incidentally discovered, COVID-related pancreatitis in a young patient with lymphoma who underwent routine restaging imaging as part of his ongoing cancer management. A few studies have been carried out previously, which demonstrate the viral attack on pancreatic cells, resulting in some form of pancreatic injury reflected either as an initial mode of presentation, i.e., acute pancreatitis or more commonly in some form of pancreatic insufficiencies such as poor glycaemic control or chronic diarrhoea [3]. Post-COVID vaccine-positive FDG findings have also been reported in the axillary lymph nodes and thyroid gland [4].

## Case presentation

A 35-year-old man was diagnosed to have a relapsed, refractory form of classical Hodgkin lymphoma in 2017, and following treatment with multiple therapies failing to control the disease, had an allogeneic stem cell transplant in 2021 with a resolution of the disease and no positive imaging findings to suggest disease relapse. Since his transplant, he was suspected to have engraftment syndrome chiefly presenting in the form of skin changes, which was well-controlled earlier in the course with topical steroids and cyclosporin (stopped after a few weeks upon clinical resolution). His only regular medications included lansoprazole and prophylactic antiviral, anti-fungal and bacterial medications, i.e., co-trimoxazole, acyclovir and fluconazole. In December 2021, he contracted COVID-19, requiring admission to the intensive therapy unit (ITU) for respiratory support with complications of pulmonary emboli and superadded Klebsiella pneumonia infection. During this inpatient admission, he developed labile blood glucose levels, which were initially attributed to intravenous steroid therapy.

**Figure 1:**
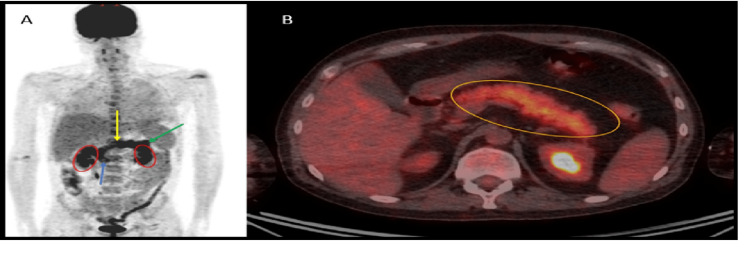
A. PET-CT planar image showing diffuse uptake in the pancreas, which is projected between the kidneys (red ovals). The blue arrow shows the pancreas head, the yellow arrow the pancreas body, and the green arrow the pancreas tail. B. Fused axial PET-CT showing the pancreas outlined by the orange oval, with diffuse FDG uptake within the pancreatic parenchyma PET: positron emission tomography; FDG: 18-fluoro-2-deoxyglucose

He was discharged home at the beginning of January 2022 and remained clinically well. However, he was again admitted to a local hospital a few days later, labelled as having COVID-19 infection (detected by COVID polymerase chain reaction (PCR)) managed with oxygen support and discharged successfully after a week. He was then brought into the clinic for his routine follow-up by the lymphoma team two weeks later and a previously scheduled whole-body 18-fluoro-2-deoxyglucose (FDG) positron emission tomography (PET) CT was performed as part of the routine restaging process. The scan confirmed ongoing complete metabolic response with no evidence of disease relapse but showed increased diffuse homogenous FDG uptake {SUV max 5.6 (3.168)} throughout the pancreas suggestive of an inflammatory process/pancreatitis (Figure 1). A detailed review of the patient's history was performed to identify possible risk factors for pancreatitis. An ultrasound scan showed no evidence of gallstones. There was no history of alcohol excess. There was no clinical history of abdominal pain and no CT evidence of pancreatic calcification to suggest prior chronic pancreatitis. Serum amylase levels were within normal limits (53 IU/L - Reference values 30-118) and lipase levels (81 U/L - Reference Values 12-53), although found to be minimally elevated, were well below the diagnostic criterion for acute pancreatitis. Virology screens for Cytomegalovirus and Ebstein Barr Virus were negative. He was clinically well with no clinical symptoms of graft versus host disease. Serum blood glucose levels remained labile consistent with diabetes, and he was commenced on insulin.

**Figure 2:**
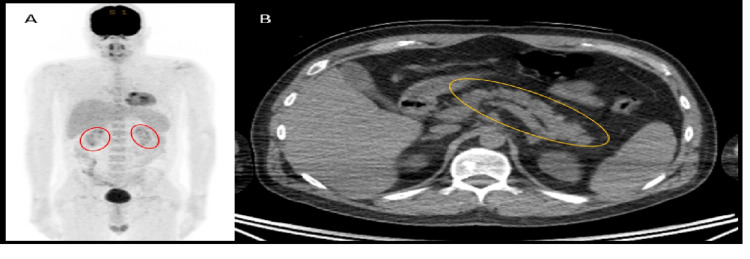
Normal PET-CT planar image six months post COVID recovery showing kidneys outlined by red oval shapes, and no abnormal FDG uptake centrally in the region of the pancreas. B. The pancreas (orange oval) has a normal morphology on CT with no calcification to suggest chronic pancreatitis PET: positron emission tomography; FDG: 18-fluoro-2-deoxyglucose

With no other source to account for the appearances in the pancreas, the tumour board felt the most likely cause of the diffuse mild FDG uptake in the pancreas was COVID-19-related pancreatitis. A routine follow-up PET-CT was performed six months later, at which point he was COVID-19 negative (tested by PCR) (Figure 2) and showed no abnormal FDG uptake in the pancreas. The patient remains diabetic, requiring insulin to modulate serum glucose levels.

## Discussion

The COVID-19 pandemic and subsequent medical research have led to the discovery of various incidental findings that highlight the non-respiratory effects of SARS-CoV-2. Radiological imaging in this regard has played a vital role in identifying these pathologies. Of these, axillary node inflammation post-vaccine injection, thyroiditis and pancreatitis have been highlighted by FDG-PET CT in otherwise asymptomatic patients. There have also been cases of patients who were otherwise asymptomatic but were found to have PET-CT findings suggesting COVID-19 infection, subsequently testing positive for SARS-CoV-19 on PCR testing. A comprehensive study carried out by the University College London Hospitals NHS Foundation Trust (UCLH) provides data about the incidental PET-CT findings of thoracic and extra-thoracic manifestations of COVID infection during the initial period of the COVID pandemic in March 2020. According to this study, 7% of patients had incidental positive findings. There is, however, limited data to fully relate these findings to COVID and rule out other causes [5].

There are also numerous case reports of incidental findings of thyroiditis in otherwise asymptomatic patients that were detected by an FDG-PET scan in patients recently positive for SARS-CoV-19. In a study that involved about 334 patients with COVID-19, it was observed that thyroid-stimulating hormone (TSH) and thyroxine values were reduced compared with baseline with no overt signs and symptoms. A few cases have been reported with typical findings of subacute thyroiditis presenting with neck pain and palpitations with ultrasound findings of an enlarged thyroid gland and diffuse hypo-echogenicity. Half of these patients became euthyroid while the remaining 50% were hypothyroid at six weeks following presentation. There have also been cases of Graves’ thyrotoxicosis in patients with COVID-19, some of whom had no previous documentation of autoimmune thyroid disease. General viral infections may not trigger the presentation of autoimmune thyroid disease; however, it has been proposed that cytokine shower initiated by SARS-CoV-2 makes it a particular trigger for autoimmune thyroid disease [6].

COVID infection is also known to affect the adrenal glands as demonstrated in several publications. Following the first COVID-19 SARS outbreak, hypercortisolism (defined as either 8 am cortisol ≤138 nmol/L, or stimulated cortisol ≤550 nmol/L following 250 mcg teracacidin) was one of the major effects observed. Adrenal insufficiency attributed to acute adrenal infarction and adrenal haemorrhage has been described in case reports following COVID-19 [4,7].

The most well-reported incidental FDG-PET finding related to COVID-19 is that of incidental FDG uptake in axillary nodes in the injected arm post-vaccine. It has historically been recognized that FDG activity on PET-CT may occur after vaccination, as has been described in numerous case reports of the influenza and HPV mass vaccination programs. Contrary to this well-established fact, numerous cases of increased uptake of FDG have been reported in cancer patients following their COVID vaccination as an incidental discovery without any clinical manifestation. A retrospective study carried out showed that about 10% of patients who had no visible axillary nodal uptake on PET imaging performed pre-vaccination, exhibited positive axillary lymph nodes after COVID-19 vaccination [8]. An analysis of 728 patients revealed the presence of hypermetabolic adenopathy in 36.4% of the patients who had received a single dose of the Pfizer-BioNTech (BNT162b2) vaccine and in 53.9% of those who had completed the vaccine schedule. In most of the cases, the lymph nodes were not pathological in size (86%) and were localized at the axillary level I ipsilateral to the injection site (99%) although lymph nodes in axillary levels II and III, interpectoral, and, less frequently, supraclavicular were also detected [9].

COVID-related pancreatitis has been reported in several studies carried out following the recent SARS-CoV-2 pandemic, most of which were either picked up on abnormal blood results in patients ending up in intensive care due to widespread infection or very rarely as an initial mode of presentation. About 51.3% of 39 nondiabetic patients diagnosed with SARS were found to have hyperglycemia meeting diagnostic criteria for diabetes during their inpatient admission [10]. There is, however, very limited data that report incidental pancreatitis identified by FDG-PET.

A few studies demonstrating the effects of the infection on the pancreatic cells had already been published based on the SARS-CoV infection from 2002-2004, before the advent of SARS-CoV-2 in 2019. A high expression of ACE surface receptors in the pancreatic islet cells makes them an optimum target for the SARS-CoV-2 virus, which then initiates a cascade of general inflammatory responses causing damage to the gland or by the direct cytopathic effects of the viral load. Recent studies show that some sort of pancreatic damage was found in most COVID-19 patients, resulting in mildly increased levels of amylase, lipase, and pathological changes in various imaging modalities [11].

A recent small study (n = 10-15 per group) from Italy has demonstrated that COVID-19 may disrupt β-cell function in patients without known diabetes. Both patients with acute COVID-19 and those recovering from COVID-19 were found to have an increased insulin response to arginine stimulation compared with healthy individuals, which suggested that COVID-19 may cause β-cell hypersecretion, which could, in turn, result in relative secretory failure [12].

In this case, the patient had a mildly complex background due to his post-stem cell transplant complication of cutaneous graft versus host disease (GvHD), however, it is well-established that it had resolved quickly with short-term steroids and cyclosporin (which were discontinued after a few weeks of initiation) before he developed pancreatitis. Moreover, neither of the other features of GvHD was noted in the patient at the time of these findings in the pancreas. Although GvHD is well-known to affect the gastrointestinal tract in the form of chronic symptoms, such as diarrhoea and malabsorption (likely to be secondary to chronic pancreatitis) [13], most cases of acute pancreatitis are almost always found in patients diagnosed to have a severe form of GvHD (Grades 3 and 4) [14], which the patient did not have at the time of diagnosis. Considering the recent novel findings of SARS-CoV-2 silently causing inflammatory findings in other endocrine organs, these findings of a mild diffuse FDG uptake in the pancreas, and a lack of pancreatic oedema or peripancreatic inflammatory change suggest the inflammatory process was mild, most likely because of the widespread immune response to the SARS-CoV-2 infection alongside the direct viral attack, further supported by the clinical exclusion of other causes. Diabetes mellitus can occur after acute/severe pancreatitis but is usually a more delayed process, occurring in up to 15% of pancreatitis patients at one year and after discharge from the hospital (Das SLM, 2014) [15].

## Conclusions

COVID continues to manifest itself in various unexpected ways, and sequelae are often discovered incidentally in medical imaging performed for non-COVID reasons. Although there have been case reports of COVID presenting as severe or acute pancreatitis and/or causing states of hyperglycemia, it is unclear whether they are directly caused by a stress response that occurs in any severe illness (characterized by increased cortisol and glucagon, resulting in a relative insulin deficiency) or by direct damage to the β-cell structure and function. Functional radiological imaging, such as FDG PET-CT, which is primarily used to investigate or stage malignant pathology, has recently gained traction for the investigation of inflammatory conditions, such as sarcoid, rheumatoid arthritis, and inflammatory bowel disease, and it may prove to have a role in the investigation and follow-up of the chronic or insidious manifestations of COVID-19.

## References

[1] Forchette L, Sebastian W, Liu T (2021). A comprehensive review of COVID-19 virology, vaccines, variants, and therapeutics. Curr Med Sci.

[2] Ai T, Yang Z, Hou H (2020). Correlation of chest CT and RT-PCR testing for coronavirus disease 2019 (COVID-19) in China: a report of 1014 cases. Radiology.

[3] Zeng W, Qi K, Ye M (2022). Gastrointestinal symptoms are associated with severity of coronavirus disease 2019: a systematic review and meta-analysis. Eur J Gastroenterol Hepatol.

[4] McIntosh LJ, Bankier AA, Vijayaraghavan GR, Licho R, Rosen MP (2021). COVID-19 vaccination-related uptake on FDG PET/CT: an emerging dilemma and suggestions for management. AJR Am J Roentgenol.

[5] Halsey R, Priftakis D, Mackenzie S (2021). COVID-19 in the act: incidental 18F-FDG PET/CT findings in asymptomatic patients and those with symptoms not primarily correlated with COVID-19 during the United Kingdom coronavirus lockdown. Eur J Nucl Med Mol Imaging.

[6] Rodríguez-Alfonso B, Ruiz Solís S, Silva-Hernández L, Pintos Pascual I, Aguado Ibáñez S, Salas Antón C (2021). 18F-FDG-PET/CT in SARS-CoV-2 infection and its sequelae. Rev Esp Med Nucl Imagen Mol (Engl Ed).

[7] Hashim M, Athar S, Gaba WH (2021). New onset adrenal insufficiency in a patient with COVID-19. BMJ Case Rep.

[8] Schroeder DG, Jang S, Johnson DR (2021). Frequency and characteristics of nodal and deltoid FDG and 11C-choline uptake on PET performed after COVID-19 vaccination. AJR Am J Roentgenol.

[9] Cohen D, Krauthammer SH, Wolf I, Even-Sapir E (2021). Hypermetabolic lymphadenopathy following administration of BNT162b2 mRNA Covid-19 vaccine: incidence assessed by [18F]FDG PET-CT and relevance to study interpretation. Eur J Nucl Med Mol Imaging.

[10] Wang F, Wang H, Fan J, Zhang Y, Wang H, Zhao Q (2020). Pancreatic injury patterns in patients with COVID-19 pneumonia. Gastroenterology.

[11] Liu F, Long X, Zhang B, Zhang W, Chen X, Zhang Z (2020). ACE2 expression in pancreas may cause pancreatic damage after SARS-CoV-2 infection. Clin Gastroenterol Hepatol.

[12] Montefusco L, Ben Nasr M, D'Addio F (2021). Acute and long-term disruption of glycometabolic control after SARS-CoV-2 infection. Nat Metab.

[13] Akpek G, Valladares JL, Lee L, Margolis J, Vogelsang GB (2001). Pancreatic insufficiency in patients with chronic graft-versus-host disease. Bone Marrow Transplant.

[14] Ko CW, Gooley T, Schoch HG (1997). Acute pancreatitis in marrow transplant patients: prevalence at autopsy and risk factor analysis. Bone Marrow Transplant.

[15] Das SL, Singh PP, Phillips AR, Murphy R, Windsor JA, Petrov MS (2014). Newly diagnosed diabetes mellitus after acute pancreatitis: a systematic review and meta-analysis. Gut.

